# Case presentation: persistent adenovirus B3 infections associated with bronchiolitis obliterans treated with cidofovir in a child with mosaic tetrasomy 9p

**DOI:** 10.1186/s12879-018-3441-x

**Published:** 2018-10-22

**Authors:** Rhiannon Ions, Manjith Narayanan, Michael Browning, Erol A. Gaillard, Gary Stiefel, Julian W. Tang

**Affiliations:** 1 Children’s Hospital, University Hospitals of Leicester, NHS Trust, Leicester, UK; 2 Infection, Immunity and Inflammation, University of Leicester, Leicester, UK; 3 Immunology, University Hospitals of Leicester, NHS Trust, Leicester, UK; 4 Clinical Microbiology and Virology, University Hospitals of Leicester NHS Trust, Level 5 Sandringham Building, Leicester Royal Infirmary, Infirmary Square, Leicester, LE1 5WW UK

**Keywords:** Case report, Adenovirus, Bronchiolitis obliterans, Mosaic tetrasomy 9p, Immune dysregulation, Cidofovir

## Abstract

**Background:**

Adenoviruses (AdV) are non-enveloped, double-stranded DNA viruses with multiple serotypes, which cause a variety of end-organ disease in both immunocompetent and immunocompromised individuals. Some adenoviruses can become latent in the mucosa-associated lymphoid tissue (e.g. adenoids and tonsils), with the potential to reactivate sporadically, leading to upper or lower respiratory tract infection and disease. Bronchiolitis Obliterans (BO) is a rare chronic lung disorder which usually follows a severe insult to the respiratory tract. In children, it is a complication of severe infections (as post-infectious BO), typically manifesting after a severe respiratory infection, in previously healthy pre-school children. Symptoms and signs of air trapping (hyperinflated chest, expiratory wheeze) with persistent oxygen requirement are characteristic. The presence of the unusual mosaic tetrasomy 9p genotype in this case, despite standard cidofovir therapy for persistent or chronic adenovirus infection, may have impacted on the child’s long-term clinical outcomes.

**Case presentation:**

We present a case of persistent AdV B3 infection in a 14-month old boy with mosaic tetrasomy 9p, which persisted for 10 weeks, resulting in radiologically-confirmed BO, requiring cidofovir to control the persistent AdV B3 infection and standard therapy with pulsed steroids. We argue that in the presence of the mosaic tetrasomy 9p, earlier antiviral therapy may have decreased the severity of BO, as this mutation is known to be associated with some degree of immune dysregulation.

**Conclusions:**

Adenovirus infections are common in children and may persist as latent infections, with subsequent reactivations during loss of immune control, related to systemic illness arising from other causes. In chronic, reactivated AdV infection with pneumonia, BO is a recognised complication. However, in this case, with the presence of the mosaic tetrasomy 9p mutation, earlier antiviral therapy may have reduced such longer term complications, due to the immune dysregulatory nature of this mutation.

## Background

Adenoviruses (AdV) are non-enveloped, double-stranded DNA viruses with multiple serotypes, which cause a variety of end-organ disease in both immunocompetent and immunocompromised individuals [1, 2]. There is growing evidence that some adenoviruses can become latent in the mucosa-associated lymphoid tissue (e.g. adenoids and tonsils), with the potential to reactivating sporadically, leading to upper or lower respiratory tract infection and disease [2, 3].

Bronchiolitis Obliterans (BO) is a rare chronic lung disorder which usually follows a severe insult to the respiratory tract [4–8]. In children, it is a common sequel of severe infections, often termed as post-infectious BO. The natural history of this condition is persistence of chronic respiratory symptoms for longer than 4–8 weeks following an acute severe respiratory infection, in children who were previously healthy. Though this can happen at any age, the incidence is highest in infancy/pre-school children. Symptoms and signs of air trapping (hyperinflated chest, expiratory wheeze) and persistent oxygen requirement are typical features.

Mosaic tetrasomy 9p is a rare somatic mutation that has been known about for the past 20 years [9, 10]. It manifests clinically with various congenital abnormalities, including severe intellectual impairment and delayed growth and dysmorphic features (e.g. hypertelorism, ear abnormalities, microretrognathia, bulbous nose, microcephaly, growth retardation, joint dislocation and scoliosis). Genetically, it is due to the presence of an additional chromosome that incorporates two copies of the 9p arm in either an isodicentric or pseudodicentric manner. In patients living with this genotype, the two forms are usually present in a mosaic state, hence the term mosaic tetrasomy 9p [11]. More recently, it has been associated with some degree of immune dysregulation [12], which may demonstrate a potential to impact on host responses to infections, though no specific immunodeficiency syndrome has yet been linked to this mutation [13].

We present a case of persistent AdV B3 infection in an infant with mosaic tetrasomy 9p, which persisted for 10 weeks, resulting in BO, requiring treatment with steroids and cidofovir, to control the persisting AdV B3 infection. We suggest that, in the presence of the mosaic tetrasomy 9p mutation, that earlier antiviral therapy may have decreased the onset and severity of the BO.

## Case presentation

A 14-month old boy presented with respiratory distress, wheeze and hypoxia. This was preceded by 24 h of coryza, fever and reduced feeding. He was admitted directly to intensive care, where he was diagnosed with pneumonia. Treatment was started with empirical antibiotics. He was intubated and ventilated for 4 days, after which he was extubated and stepped down to high dependency unit (HDU) on continuous positive airways pressure (CPAP) ventilation. He continued to have persistently increased work of breathing, persistent expiratory wheeze and symptoms and signs of air trapping.

Though he did not progress to respiratory failure, weight gain and oxygenation was achieved only by initiation of Heated Humidified High Flow Nasal cannula therapy (HHHFNC).

Polymerase chain reaction (PCR) testing of nasopharyngeal aspirates (NPAs) revealed the persistence of rhinovirus and adenovirus for 10 weeks in both NPAs and bronchoalveolar lavages (BALs), with parainfluenza type 3 found in just one NPA sample. Adenovirus was also found in blood by PCR testing. Rhinovirus is not normally tested for in blood samples and there was no validated assay available for this. One BAL and one urine sample were screened for cytomegalovirus (CMV) by PCR also, and found to be negative. One stool sample was tested and found to be negative for rotavirus and adenovirus (despite the persistence of adenovirus in the respiratory samples at this time).

There was no significant family history of any genetic diseases and the patient was born at term. Mum is a smoker but states that she did not smoke during her pregnancy. During pregnancy, intrauterine growth restriction was identified. At the time of admission his weight was < 0.4th centile, but this was increasing. His parents report poor feeding since birth and an increased work of breathing from 5 months old. He is known to the allergy services for severe eczema and faltering weight, and has been diagnosed with cow’s milk protein allergy. His eczema medications have included topical tacrolimus, moderately potent topical steroids and emollient therapy.

His other medical history includes two previous episodes of bronchiolitis, three and 1 month prior to this admission (including one overnight stay during a trip to Australia). Prior to admission, he had only received two courses of antibiotics in his life – once for an infected BCG vaccine site and once for an ear infection.

**Fig. 1:**
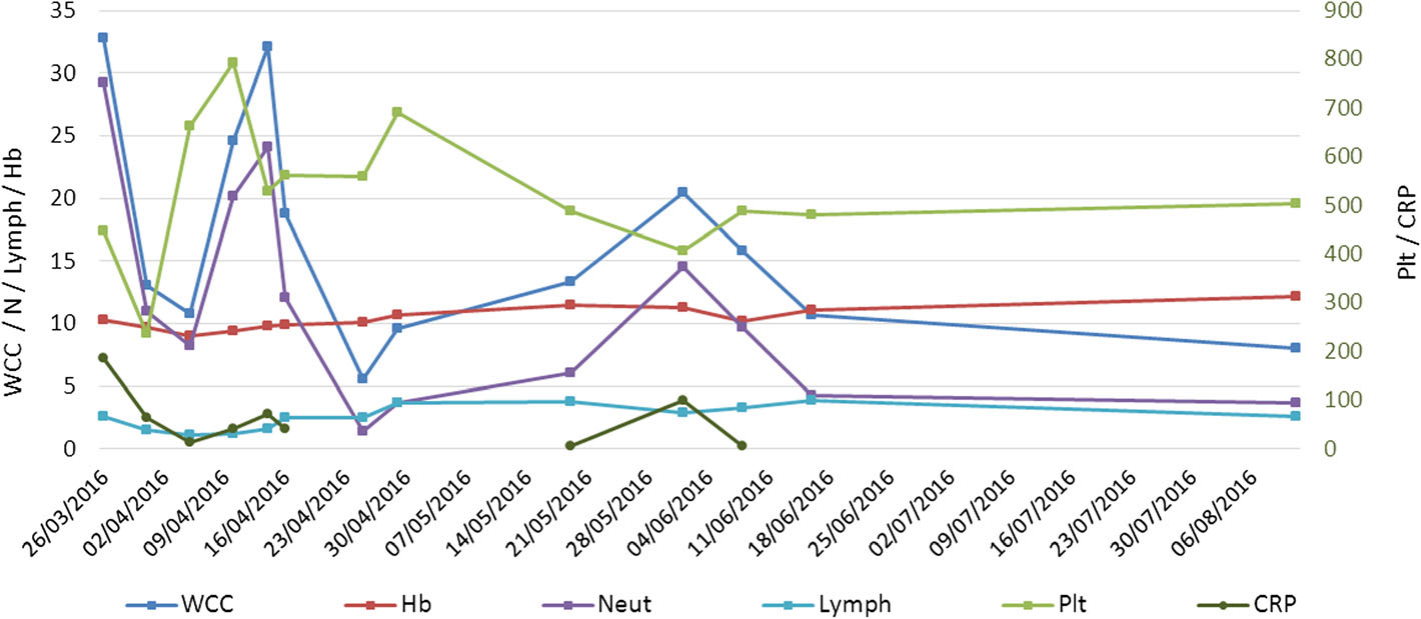
Plots of the child’s blood parameters showing their fluctuating values during the adenovirus infection period. WCC – white cell count (× 10^9^/L); Hb – haemoglobin (g/dL); Neut – neutrophils (× 10^9^/L); Lymph – lymphocytes (× 10^9^/L); Plt – platelets (× 10^9^/L); CRP – C-reactive protein (mg/L)

During his inpatient stay, he had 2 further intercurrent bacterial respiratory infections, treated by broad spectrum antibiotics. Immunological investigations revealed an IgG and IgM hypogammaglobunemia but a normal IgA. He was up-to-date with his childhood immunisations prior to admission, but has had a poor response to tetanus and twice to haemophilus B, demonstrated by low IgG levels to both. He was also found to have near absent B-cells and a reduced number of T-cells, and prophylaxis for *Pneumocystis jiroveci* was commenced. HIV testing was negative on two occasions. We withheld any further live virus vaccinations, in view of his immunodeficiency (Table 1**,** Fig. 1).

**Table 1:** Immunological results at presentation

Serology	Patient results	Age-related normal range
IgG	1.8 g/L	3.0–9.0
IgA	0.33 g/L	0.20–0.70
IgM	0.42 g/L	0.60–2.1
Anti-tetanus toxoid	0.03 IU/ml	> 0.15 (protective level)
Anti-Haemophilus type B	< 0.11 mg/L	> 1.0 (protective level)
Lymphocyte subsets		
Absolute lymphocyte count	1.04 × 10^9^/L	3.4–9.0
CD3+ (T cells)	0.95 × 10^9^/L	1.90–5.9
CD3 + CD4+ (helper T)	0.74 × 10^9^/L	1.40–4.3
CD3 + CD8+ (cytotoxic T)	0.20 × 10^9^/L	0.50–1.70
CD16 + CD56+ (NK cells)	0.03 × 10^9^/L	0.16–0.95
CD19+ (B cells)	0.01 × 10^9^/L	0.61–2.60

**Fig. 2:**
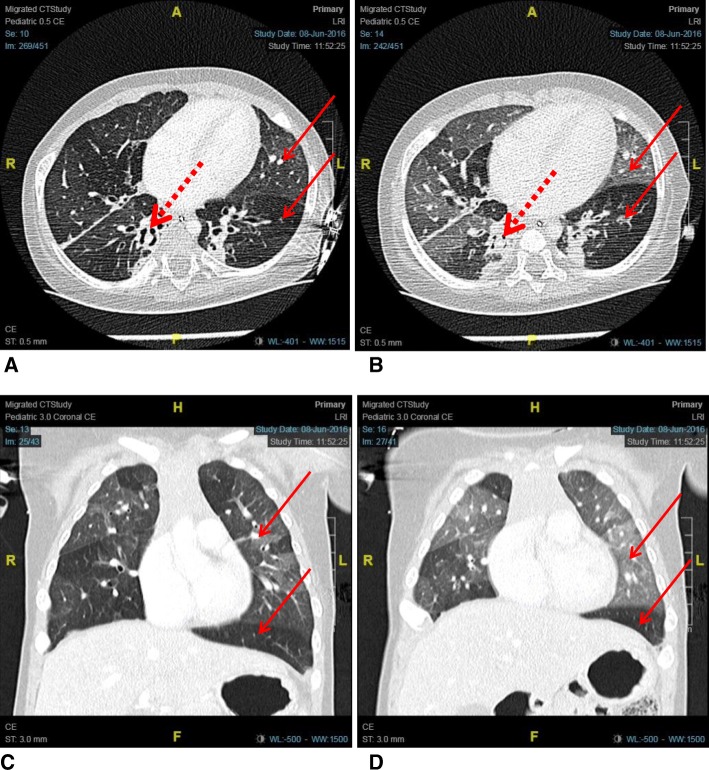
**a** Inspiratory cross-sectional; **b** Expiratory cross-sectional; **c** Inspiratory coronal; **d** Expiratory coronal. Areas of hypo- and hyper-attenuation (thin arrows), which are more evident on expiratory scans, showing mosaic perfusion. Traction bronchiectasis is also shown (thick dotted arrows). The images show features recognised to be associated with bronchiolitis obliterans, including: mosaic perfusion, air trapping, vascular attenuation, bronchiectasis and peribronchial thickening, as seen in the mosaic pattern of attenuation of both lung fields

At this stage, a CT chest was performed (Fig. 2). The findings (mosaic pattern attenuation of both lung fields with a combination of air trapping and oligaemia, more pronounced in expiratory images) were consistent with BO which was believed to be secondary to the persistent adenovirus rather than the rhinovirus infection. Reasons for this were mainly that rhinoviruses are predominantly upper respiratory tract infections, whereas AdVs can cause more systemic infections and AdV DNA was detected in the blood. No biopsy or histopathological investigation was performed in order to confirm BO.

A flexible bronchoscopy showed structurally normal airways. Screening for tuberculosis was negative.

After a multidisciplinary team discussion, it was decided to commence pulse methyl-prednisolone therapy (3 daily doses of 10 mg/kg given every month) and long-term azithromycin (10 mg/kg 3 doses every week) for management of BO. Prior to commencing the steroids, cidofovir was administered to control the adenovirus replication and reduce the dissemination of the adenovirus.

Cidofovir (5 mg/kg, intravenously, with probenecid) was given on fortnightly basis for 4 months, during which he his renal function was monitored weekly. All the patient’s renal function test results remained within our institutional laboratory’s normal ranges (sodium 133–146 mmol/L; potassium 3.5–5.0 mmol/L; urea 1.5–5.0 mmol/L; creatinine 15–31 μmol/L) throughout the treatment period with cidofovir, indicating good tolerance to the drug. Since commencing steroid therapy, it was possible to gradually wean him off his HHHFNC, and he has since been thriving well. He was discharged on supplemental oxygen, which was weaned off over the next 12 months.

An array comparative genomic hybridisation (CGH) revealed mosaic tetrasomy of short arm of chromosome 9. There are no reports yet of immunodeficiency in association with mosaic tetrasomy 9p, nor any specific vulnerability to any particular viral infections [13], but has previously been associated with auto-immune conditions associated with IFN-1 dysregulation (predisposing to inflammatory myositis and lupus-like syndrome) [12].

A conclusive diagnosis of a specific immunodeficiency/immunomodulation is yet to be made in this case, as this profile does not fit any primary immune deficiency. Molecular genetic testing for mutations in 82 genes associated with primary immunodeficiency and gastrointestinal disorders (TIGER panel, Great Ormond Street Hospital, London) did not identify any clearly pathogenic variants. The patient is currently being given weekly supplemental immunoglobulins.

Most recently, the child has now cleared the long-term rhinovirus and adenovirus infections, and has been weaned off steroids and long term oxygen therapy. His weight gain has been adequate on air, and he is still too young for spirometry testing. Ongoing problems include recent *Pseudomonas aeruginosa* infection, with a wet cough, which is likely due to the bronchiectasis component of the BO disease.

## Discussion and conclusion

This case demonstrates the long-term shedding (for 10 weeks) of rhinovirus and Adv B3, as detectable by the PCR testing of respiratory secretions, in a young boy who developed post-adenoviral bronchiolitis obliterans on the background of a potential, as yet undefined, immunodeficiency and chromosomal anomaly (mosaic tetrasomy 9p).

It is likely that chronic AdV B3 infection contributed to persistent inflammation which may have contributed to the development of BO. This may have been further exacerbated by a dysregulated IFN-1 response, which has been described previously in association with tetrasomy 9p [12]. The relatively benign course and response to steroid therapy suggests a mild disease, and it is possible that cidofovir therapy acted as a disease modifying agent by reducing the viral load and associated immune stimulus during the active inflammation phase.

Adenovirus B3 is well-recognised to cause respiratory infections, particularly in children [1, 2, 4]. Persistent adenovirus infections with latency in children have been described [2, 3]. Post-infective BO is an uncommon complication of adenoviral infection [4–8].

In particular, Chung et al. [4] describe nine severe cases of adenovirus infection, all of whom had severe symptoms – similar to the case described here. Of these nine cases, one died and five of the survivors went on to develop BO. None of these patients appeared to have been treated with cidofovir or other antiviral drugs. In a case control study of 109 consecutive cases from Argentina with BO (compared against a control population of bronchiolitis without BO), Colom et al. [8] suggested a link between severity of bronchiolitis and progression to BO. The odds ratio of children on mechanical ventilation with bronchiolitis who then went on to develop BO was 11 on multivariate regression analysis.

The general consensus among pediatric pulmonologists is that lung biopsy is now not usually required for the diagnosis of post-infectious BO [14], with chest HRCT being the preferred investigation, currently [15].

Cidofovir is an antiviral used against DNA viruses – particularly human herpesviruses and adenoviruses. It acts by binding irreversibly to the viral DNA polymerase, as a false substrate, inhibiting viral replication, and is therefore virostatic not virucidal by this mechanism of action. It is given intravenously, and needs to be given with probenecid to reduce its nephrotoxicity. Adverse effects include mainly nephrotoxicity, but also nausea and vomiting, headache, neutropenia, hair loss, eye problems (uveitis, iritis) – and more rarely deranged liver function and anemia. Although there have been no formal clinical trials of cidofovir or its related lipid-soluble form (Brincidofovir), numerous case reports/series and reviews have supported its use in transplant/immunosuppressed paediatric patients with adenovirus infections [16–21].

It is possible that cidofovir therapy can mitigate the progression to bronchiolitis obliterans by reducing the viral load. As far as we are aware cidofovir therapy has not been reported, specifically, to decrease risk of BO in children with severe adenoviral lung infection. However, since cidofovir is well-known to reduce the viral load of AdV in AdV-infected immunocompromised children [19], it is not unreasonable to propose that earlier treatment with cidofovir, in a patient with some immune dysregulation resulting from the presence of the mosaic tetrasomy 9p mutations, should reduce onset and severity of adenoviral infection and the risk of developing BO.

Adenovirus infections are common in children and may persist as latent infections, with subsequent reactivations during loss of immune control, related to systemic illness arising from other causes. In chronic, reactivated AdV infection with pneumonia, teams should be aware that BO is one of the possible complications. In the presence of somatic host mutations that may give rise to some immune dysregulation, like mosaic tetrasomy 9p, earlier treatment chronic AdV infections with antiviral drugs may reduce this risk. Further studies would be useful, but may be difficult with such rare genetic mutations.

## Abbreviations

AdV: Adenovirus
BAL: Bronchoalveolar lavage
BO: Bronchiolitis obliterans
CGH: Comparative genomic hybridisation
CPAP: Continuous positive airways pressure
CT: Computed tomography
HDU: High dependency unit
HHHFNC: Heated Humidified High Flow Nasal cannula therapy
NPA: Nasopharyngeal aspirate
PCR: Polymerase chain reaction

## References

[1] James L, Vernon MO, Jones RC, Stewart A, Lu X, Zollar LM (2007). Outbreak of human adenovirus type 3 infection in a pediatric long-term care facility--Illinois, 2005. Clin Infect Dis.

[2] Kalu SU, Loeffelholz M, Beck E, Patel JA, Revai K, Fan J (2010). Persistence of adenovirus nucleic acids in nasopharyngeal secretions: a diagnostic conundrum. Pediatr Infect Dis J.

[3] Garnett CT, Talekar G, Mahr JA, Huang W, Zhang Y, Ornelles DA (2009). Latent species C adenoviruses in human tonsil tissues. J Virol.

[4] Chuang Yu CCH, Wong KS, Huang JG, Huang YC, Chang LY (2003). Severe adenovirus infection in children. J Microbiol Immunol Infect.

[5] Jeon JS, Yi HA, Ki SY, Jeong SW, Uh ST, Jin SY (1997). A case of bronchiolitis obliterans organizing pneumonia associated with adenovirus. Korean J Intern Med.

[6] de Blic J, Deschildre A, Chinet T. [Post-infectious bronchiolitis obliterans]. Rev Mal Respir 2013;30:152–160. [Article in French]. 10.1016/j.rmr.2012.10.600 23419446

[7] Li YN, Liu L, Qiao HM, Cheng H, Cheng HJ (2014). Post-infectious bronchiolitis obliterans in children: a review of 42 cases. BMC Pediatr.

[8] Colom AJ, Teper AM, Vollmer WM, Diette GB (2006). Risk factors for the development of bronchiolitis obliterans in children with bronchiolitis. Thorax.

[9] Stumm M, Tönnies H, Mandon U, Götze A, Krebs P, Wieacker PF (1999). Mosaic tetrasomy 9p in a girl with multiple congenital anomalies: cytogenetic and molecular-cytogenetic studies. Eur J Pediatr.

[10] Eggermann T, Rossier E, Theurer-Mainka U, Backsch C, Klein-Vogler U, Enders H (1998). New case of mosaic tetrasomy 9p with additional neurometabolic findings. Am J Med Genet.

[11] El Khattabi L, Jaillard S, Andrieux J, Pasquier L, Perrin L, Capri Y (2015). Clinical and molecular delineation of Tetrasomy 9p syndrome: report of 12 new cases and literature review. Am J Med Genet A.

[12] Frémond ML, Gitiaux C, Bonnet D, Guiddir T, Crow YJ, de Pontual L (2015). Mosaic Tetrasomy 9p: a Mendelian condition associated with pediatric-onset overlap myositis. Pediatrics.

[13] Genetic and Rare Diseases Information Centre (GARD). Tetrasomy 9p. https://rarediseases.info.nih.gov/diseases/42/tetrasomy-9p. Accessed 14 Jun 2018.

[14] Chang AB, Masel JP, Masters B (1998). Post-infectious bronchiolitis obliterans: clinical, radiological and pulmonary function sequelae. Pediatr Radiol.

[15] Champs NS, Lasmar LM, Camargos PA, Marguet C, Fischer GB, Mocelin HT (2011). Post-infectious bronchiolitis obliterans in children. J Pediatr (Rio J).

[16] Carter BA, Karpen SJ, Quiros-Tejeira RE, Chang IF, Clark BS, Demmler GJ (2002). Intravenous Cidofovir therapy for disseminated adenovirus in a pediatric liver transplant recipient. Transplantation.

[17] Neofytos D, Ojha A, Mookerjee B, Wagner J, Filicko J, Ferber A (2007). Treatment of adenovirus disease in stem cell transplant recipients with cidofovir. Biol Blood Marrow Transplant.

[18] Lindemans CA, Leen AM, Boelens JJ (2010). How I treat adenovirus in hematopoietic stem cell transplant recipients. Blood.

[19] Matthes-Martin S, Feuchtinger T, Shaw PJ, Engelhard D, Hirsch HH, Cordonnier C (2012). European guidelines for diagnosis and treatment of adenovirus infection in leukemia and stem cell transplantation: summary of ECIL-4 (2011). Transpl Infect Dis.

[20] Lopez SMC, Michaels MG, Green M (2018). Adenovirus infection in pediatric transplant recipients: are effective antiviral agents coming our way?. Curr Opin Organ Transplant.

[21] Yusuf U, Hale GA, Carr J, Gu Z, Benaim E, Woodard P (2006). Cidofovir for the treatment of adenoviral infection in pediatric hematopoietic stem cell transplant patients. Transplantation.

